# Factors affecting cost-related medication non-adherence among US population with cardiovascular risk factors

**DOI:** 10.1017/S1463423626101054

**Published:** 2026-03-27

**Authors:** Nikhila Gandrakota, Manju Ramakrishnan, Kavya Sudireddy, Megha K. Shah

**Affiliations:** 1 Emory University School of Medicine, Atlanta, USA; 2 Cleveland Clinic, Cleveland, Ohio, USA; 3 Rollins School of Public Health: Emory University Rollins School of Public Health, USA; 4 UMass Chan Medical School TH Chan School of Medicine: University of Massachusetts, USA; 5 Emory University School of Medicine, USA

**Keywords:** Cardiovascular, cost, medication, non-adherence, risk factors

## Abstract

**Introduction:**

In the US, cardiovascular diseases (CVD) are the leading cause of death and disability. Cost-related medication non-adherence (CRMN) can have serious consequences and worsen CVD outcomes. We examined the relationship between CVD risk factors and CRMN among US adults.

**Methods:**

CDC’s 2019–2021 National Health Interview Survey (NHIS) data were used to examine CRMN among adults, categorized into three groups based on reported risk factors. We used chi-square tests, and logistic regression to determine factors associated with CRMN.

**Results:**

Among 49,464 participants, young, unmarried individuals, females, less educated, and participants from the South had higher CRMN than older, married individuals, males, and those with higher education residing in the other regions. Current smokers and those with more CVD risk factors also reported higher CRMN than former and never-smokers. Conversely, those aged 65 or older, with high-income, and excellent self-rated health had lower CRMN than younger participants, low-income families, and those with poor self-rated health. Public insurance and Medicaid participants had lower CRMN than uninsured (OR 0.13, 95% CI, 0.04–0.45, and OR 0.24, 95% CI, 0.15–0.36). Stratified analysis by diabetes, hypertension, and hyperlipidemia, revealed participants with high-income had lower odds of CRMN (OR 0.38, 95% CI, 0.28–0.50; OR 0.39, 95% CI, 0.28–0.58; OR 0.37, 95% CI, 0.27–0.51 respectively) than those with lower- incomes.

**Conclusion:**

Adults under 65 with more CVD risk factors and lacking insurance coverage are at higher risk of CRMN. Therefore, strengthening prescription drug coverage and targeted interventions are necessary to reduce CRMN among those with cardiovascular risk factors.

## Introduction

Medication non-adherence tremendously impacts the healthcare system and costs the US healthcare system $500 billion per year (Chisholm-Burns and Spivey, 2012; Klein, 2020; Phillion, 2022). The costs incurred due to complications, hospitalizations, and further medical interventions resulting from the inability to take prescribed medication lead individuals into a medico-economic decline (Gourzoulidis *et al.*, 2017; Hamrahian, 2020). Several factors are responsible including the high cost of medications, long-term treatment regimens, polypharmacy, insurance coverage, and lack of awareness (Kleinsinger, 2018). Chronic diseases like hypertension and diabetes often require the administration of complex multimodal medication regimens, and non-adherence can lead to disease exacerbation (Burnier and Egan, 2019; Patel *et al.*, 2019). Lack of interventions to alleviate concerns about adherence to medications can lead to poor health outcomes (Walsh *et al.*, 2019).

In the United States, non-adherence afflicts >60% of patients diagnosed with CVD, the leading cause of morbidity and mortality worldwide (Baroletti and Dell’Orfano, 2010; Lochner and Cox, 2012). Additionally, the financial burden of CVD is projected to double and reach $1.1 trillion in 2035 (Khavjou *et al.*, 2016). At the forefront of this effect are risk factors of CVDs such as sociodemographic characteristics, tobacco smoking, physical inactivity, hypertension, diabetes, and dyslipidaemia which if undermanaged, result in both the progression of the disease and excessive utilization of healthcare resources (Teo and Rafiq, 2021). Several other recent studies demonstrated low adherence rates in 10% and 11.9% of subjects on anti-hypertensive and lipid-lowering agents respectively (Hennein *et al.*, 2017). In addition, Osborn et al. showed the significance of financial strain on non-adherence rates, highlighting the need to further evaluate cost-related medication non-adherence (CRMN) in the progression of CVDs (Osborn *et al.*, 2017). In our study, to better understand and identify the people at risk for CRMN, we intend to explore the circumstances and characteristics of the population that can drive the same.

Through the analysis of a large nationally representative sample data of individuals, we describe sociodemographic, regional, and health-related factors influencing CRMN among those with cardiovascular risk factors. The study aims to determine the underlying factors among adults with CVD risk factors to better inform what groups might be at risk for cost-related non-adherence.

## Methods

We conducted a cross-sectional analysis of pooled data from the CDC’s NHIS from 2019–2021. The NHIS is an annual cross-sectional survey that collects information on the health and health-related behaviours of the U.S. population (‘NHIS - About the National Health Interview Survey’, 2023). The National Center for Health Statistics (NCHS) oversees the NHIS data collection.

Adults aged 18 years and older who reported having at least one of three CVD risk factors: diabetes, hypertension, or hyperlipidaemia, and who had received prescription medication in the past 12 months were included in the study. Participants were classified as having no, one, or more than one CVD risk factor based on their responses to whether they had ever been told they had diabetes, high blood pressure, or high cholesterol in the survey. Individuals with no CVD risk factors were included as a comparison group to examine how CRMN varies between those with and without CVD-related conditions. Many of these individuals use prescription medications for other acute or chronic health needs (e.g., mental health conditions, infections, or reproductive care), making CRMN a relevant outcome for this group as well.

CRMN was assessed by questions that asked participants whether they had skipped medication doses to save money, taken less medicine to save money, or delayed filling a prescription to save money in the past 12 months. Participants were classified as having CRMN if they answered ‘yes’ to any of these questions.

Sociodemographic characteristics of the respondents, including age, sex, education, marital status, income ratio to poverty level, insurance status, obesity status, smoking status, self-rated health status, and the number of adults and children in the household, were also assessed for the study. Chi-square tests were used as a preliminary step to explore bivariate associations between CRMN and individual sociodemographic and health-related variables. These tests provided *p*-values for unadjusted differences in CRMN prevalence across subgroups and offered descriptive context for interpreting unadjusted relationships. They also informed the selection of covariates for the performance of multivariate logistic regression models. Logistic regression was the primary analytic method used to assess independent associations.

Appropriate survey weights provided by the NHIS were applied to generate national estimates. Descriptive statistics were obtained using the percentage of cost-related non-adherence across different demographic and cardiovascular risk factor categories, including age, sex, ethnicity, educational status, income-to-poverty ratio, and marital status. Logistic regression analysis examined the association between CVD risk factors and cost-related medication non-adherence, adjusting for sociodemographic variables. Further, multivariate logistic regression analyzes were also conducted by disease condition.

All statistical analyzes were performed using SAS software, version 9.4 (SAS Institute, North Carolina, US) – a *p*-value less than 0.05% was considered significant.

## Results

### Demographics (Table 1)

**Table 1.** Characteristics of the study population with and without CRMN by risk factor status, (*N* = 143,685,241) (*n* = 49,464)

	Cost-related non-adherence, weighted % (standard error) NHIS 2019–2021						
	No CVD risk factors		Any one CVD risk factor		>1 CVD risk factor		
	Has CRMN	No CRMN	Has CRMN	No CRMN	Has CRMN	No CRMN	Chi-square *P*-value
Age							
18–39	9.0 (0.4)	91.0 (0.4)	12.5 (0.9)	87.5 (0.9)	16.1 (1.9)	83.9 (1.9)	<0.0001
40–64	7.6 (0.4)	92.4 (0.4)	8.9 (0.4)	91.1 (0.4)	11.4 (0.6)	88.6 (0.6)	
≥65	3.4 (0.4)	96.6 (0.4)	3.6 (0.3)	96.4 (0.3)	4.4 (0.3)	95.6 (0.3)	
Sex							
Male	6.6 (0.4)	93.4 (0.4)	6.5 (0.4)	93.5 (0.4)	6.7 (0.5)	93.3 (0.5)	0.0044
Female	8.6 (0.3)	91.4 (0.3)	8.9 (0.4)	91.1 (0.4)	9.8 (0.5)	90.2 (0.5)	
Ethnicity							
Non-Hispanic White	6.8 (1.3)	93.2 (0.3)	7.2 (0.3)	92.8 (0.3)	7.3 (0.4)	92.7 (0.4)	0.1666
Non-Hispanic Black	12.2 (1.1)	87.8 (1.1)	8.9 (0.8)	91.1 (0.8)	11.2 (1.1)	88.8 (1.1)	
Non-Hispanic Asian	6.4 (1.0)	93.6 (1.0)	3.5 (0.9)	96.5 (0.9)	4.3 (1.1)	95.7 (1.1)	
Non-Hispanic All other race group	10.3 (1.6)	89.7 (1.6)	8.4 (2.2)	91.6 (2.2)	14.1 (2.5)	85.9 (2.5)	
Hispanic	10.3 (0.8)	89.7 (0.8)	11.7 (1.0)	88.3 (1.0)	11.2 (1.2)	88.8 (1.2)	
Educational status							
<High school	11.1 (1.0)	88.9 (1.0)	11.7 (1.1)	88.3 (1.1)	10.4 (1.0)	89.6 (1.0)	<0.0001
College graduate	5.3 (0.3)	94.7 (0.3)	5.2 (0.4)	94.8 (0.4)	5.9 (0.5)	94.1 (0.5)	
High school grad	8.9 (0.6)	91.1 (0.6)	7.8 (0.6)	92.2 (0.6)	7.9 (0.7)	92.1 (0.7)	
Some college	9.0 (0.4)	91.0 (0.4)	9.1 (0.5)	90.9 (0.5)	9.5 (0.7)	90.5 (0.7)	
Income to poverty ratio							
<1	13.9 (1.0)	86.1 (1.0)	14.8 (1.4)	85.2 (1.4)	13.9 (1.0)	86.1 (1.0)	<0.0001
1–1.99	12.5 (0.6)	87.5 (0.6)	11.6 (0.6)	88.4 (0.6)	12.5 (0.6))	87.5 (0.6)	
≥2	4.7 (0.2)	95.3 (0.2)	4.8 (0.3)	95.2(0.3)	4.7 (0.2)	95.2 (0.4)	
Marital status							
Married	7.1 (0.3)	92.9 (0.3)	7.2 (0.3)	92.8 (0.3)	7.9 (0.5)	92.1 (0.5)	0.0029
Unmarried	8.8 (0.4)	91.2 (0.4)	8.9 (0.5)	91.1 (0.5)	8.7 (0.5)	91.3 (0.5)	
Region							
Midwest	8.2 (0.5)	91.8 (0.5)	7.6 (0.6)	92.4 (0.6)	7.7 (0.6)	92.3 (0.6)	0.0068
Northeast	5.3 (0.5)	94.7 (0.5)	5.5 (0.6)	94.5 (0.6)	5.5 (0.7)	94.5 (0.7)	
South	9.2 (0.5)	90.8 (0.5)	9.6 (0.5)	90.4 (0.5)	9.9 (0.6)	90.1 (0.6)	
West	7.4 (0.5)	92.6 (0.5)	6.7 (0.5)	93.3 (0.5)	7.9 (0.7)	92.1 (0.7)	
Insurance							
Medicaid	9.4 (0.8)	90.6 (0.8)	9.4 (1.0)	90.6 (1.0)	8.8 (0.9)	91.2 (0.9)	Unable to perform*
Medicare	6.6 (0.9)	93.4 (0.9)	6.3 (0.6)	93.7 (0.6)	7.2 (0.6)	92.8 (0.6)	
Uninsured	26.1 (1.6)	73.9 (1.6)	25.9 (2.0)	74.1 (2.0)	30.1 (3.2)	69.9 (3.2)	
Other public	6.9 (4.7)	93.1 (4.7)	0.0 (0.0)	100.0 (0.0)	3.2 (1.9)	96.8 (1.9)	
Private	5.9 (0.2)	94.1 (0.2)	6.3 (0.3)	93.7 (0.3)	7.0 (0.4)	93.0 (0.4)	
Smoking status							
Current smoker	14.8 (0.9)	85.2 (0.9)	14.5 (1.0)	85.5 (1.0)	15.9 (1.3)	84.1 (1.3)	0.0192
Former smoker	8.3 (0.5)	91.7 (0.5)	7.5 (0.5)	92.5 (0.5)	7.1 (0.5)	92.9 (0.5)	
Never smoker	6.5 (0.3)	93.5 (0.3)	6.5 (0.3)	93.5 (0.3)	7.4 (0.4)	92.6 (0.4)	
Obesity							
<18.5	5.2 (1.3)	94.8 (1.3)	9.8 (2.5)	90.2 (2.5)	3.2 (2.3)	96.8 (2.3)	<0.0001
18.5–24.9	7.0 (0.4)	93.0 (0.4)	6.6 (0.5)	93.4 (0.5)	6.5 (0.8)	93.5 (0.8)	
25–29.9	7.3 (0.4)	92.7 (0.4)	7.2 (0.5)	92.8 (0.5)	6.8 (0.5)	93.2 (0.5)	
≥30	10.2 (0.6)	89.8 (0.6)	9.0 (0.5)	91.0 (0.5)	10.1 (0.6)	89.9 (0.6)	
Self-rated health							
Excellent	4.3 (0.4)	95.7 (0.4)	3.2 (0.4)	96.8 (0.4)	2.8 (0.7)	97.2 (0.7)	<0.0001
Very good	6.7 (0.4)	93.3 (0.4)	5.0 (0.4)	95.0 (0.4)	4.7 (0.5)	95.3 (0.5)	
Good	10.5 (0.6)	89.5 (0.6)	8.3 (0.4)	91.7 (0.4)	7.3 (5)	92.7 (0.5)	
Fair	17.6 (1.4)	82.4 (1.4)	16.4 (1.0)	83.6 (1.0)	13.3 (0.9)	86.7 (0.9)	
Poor	17.6 (2.4)	82.4 (2.4)	20.3 (2.4)	79.7 (2.4)	21.1 (2.1)	78.9 (2.1)	
Count of adults							
1	10.1 (0.4)	89.9 (0.4)	7.9 (0.4)	92.1 (0.4)	8.5 (0.5)	91.5 (0.5)	0.2934
2	7.0 (0.3)	93.0 (0.3)	7.3 (0.3)	92.7 (0.3)	7.4 (0.4)	92.6 (0.4)	
3+	8.1 (0.6)	91.9 (0.6)	8.9 (0.8)	91.1 (0.8)	10.1 (1.0)	89.9 (1.0)	
Count of children							
0	7.2 (0.3)	92.8 (0.3)	7.1 (0.3)	92.9 (0.3)	7.3 (0.4)	92.7 (0.4)	<0.0001
1	8.7 (0.7)	91.3 (0.7)	10.3 (0.9)	89.7 (0.9)	11.3 (1.5)	88.7 (1.5)	
2	8.0 (0.7)	92.0 (0.7)	9.3 (1.0)	90.7 (1.0)	11.9 (1.7)	88.1 (1.7)	
3+	10.6 (1.1)	89.4 (1.1)	9.2 (1.3)	90.8 (1.3)	20.9 (3.9)	79.1 (3.9)	
Usual place of care							
Yes	7.4 (0.3)	92.6 (0.3)	7.5 (03)	92.5 (0.3)	8.1 (0.3)	91.9 (0.3)	<0.0001
No	13.6 (1.3)	86.4 (1.3)	16.4 (2.2)	83.6 (2.2)	19.9 (4.1)	80.1 (4.1)	

Unable to perform Chi-square test due to insufficient *N* in other public category in one CVD risk factor group.

In our study among a total of 49,464 participants (*N* weighted = 143,685,241) in the sample, we examined the proportion of CRMN among various demographic groups based on the CVD risk factor status which is shown in Table 1. We found that 25.37% had no CVD risk factors, 41.93% had one risk factor, and 32.79% had more than one risk factor.

**Figure 1.**
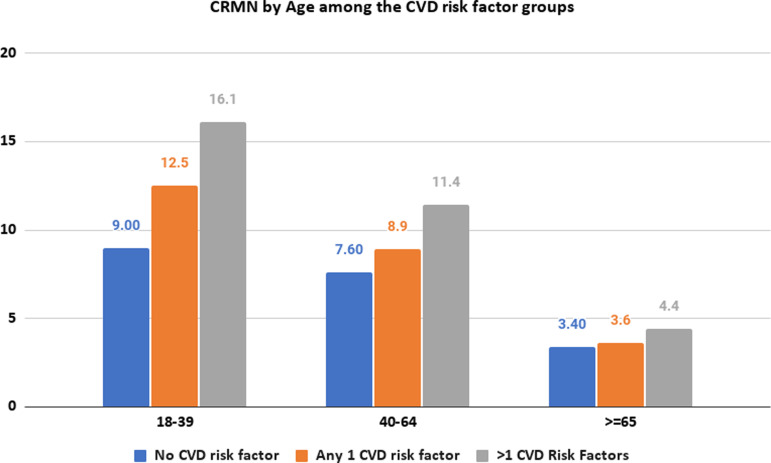
CRMN by age among the CVD risk factor groups.

In participants with a history of more than one CVD risk factor, 16.1% among those who were aged between 18–39 years, demonstrated CRMN while only 4.4% of the elderly population with similar CVD risk factor profile aged 65 years and above were non-adherent to medications. Patients with no CVD risk factors demonstrated a lower proportion of individuals exhibiting CRMN with 9% who fall within the age group of 18–39 years while 7.6% and 3.4% are between 40–65 years of age and over 65 years respectively. The differences in proportions were statistically significant (*p* < 0.0001) (Figure 1).

**Figure 2.**
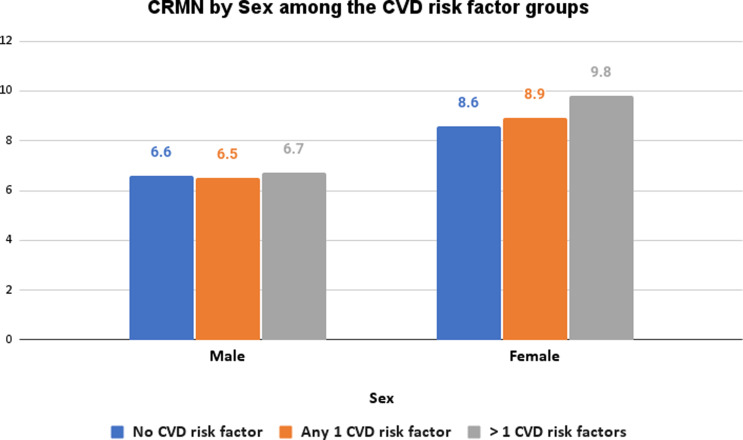
CRMN by Sex among the CVD risk factor groups.

Females had a notably higher proportion of non-adherence irrespective of their risk factor status. Specifically, 8.6% of females with no CVD risk factors and 8.9%, and 9.8% with one and more than one CVD risk factor respectively compared to 6.6% of males with no CVD risk factors, 6.5% with one risk factor, and 6.7% with more than one CVD risk factor who showed non-adherence (Figure 2).

The level of education had a significant impact on the prevalence of CRMN among participants. Individuals with less than a high school level of education had a higher proportion of non-adherence than college graduates, with 11.1%, 11.7%, and 10.4% among those with no risk factors, one risk factor, and more than one risk factor, respectively (*p* = 0.0001). Married respondents had lower non-adherence rates than unmarried respondents, with rates of 7.1%, 7.2%, and 7.9% for married patients with no risk factors, one risk factor, and more than one risk factor, respectively. Higher proportions of unmarried individuals were prone to be non-adherent. 8.8% of the unmarried respondents with no CVD risk factors displayed CRMN, with similar percentages of 8.9% and 8.7% in individuals with one and more than one CVD risk factor correspondingly (*p* = 0.0029).

**Figure 3.**
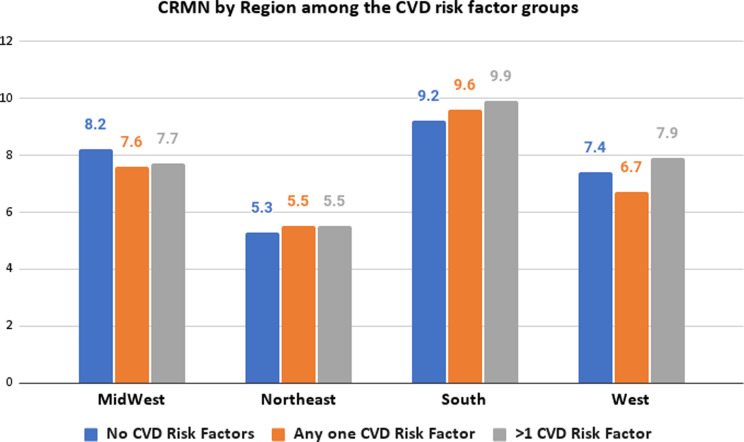
CRMN by Region among the CVD risk factor groups.

The South exhibited the highest non-adherence in patients with no risk factor, one, and multiple CVD risk factors, at 9.2%, 9.6%, and 9.9%, respectively compared to all other regions across the country. These regional differences across the country were statistically significant (*p* = 0.0068) (Figure 3). Furthermore, the results also showed that a higher prevalence of CRMN was observed in uninsured patients across all risk factor groups with 26.1%, 25.9%, and 30.1% among those with no risk factors, one risk factor, and more than one risk factor respectively.

Among respondents with more than one CVD risk factor and body mass index (BMI) greater than 30, there was a significant proportion (*p* < 0.0001) who were not adherent to medications (10.1%). Respondents who rated their health as fair, or poor had higher non-adherence (*p* < 0.0001) across all risk factor groups. Respondents with three or more children were significantly more likely to be non-adherent than those without children (*p* < 0.0001) across all risk factor groups. In addition, there was a higher prevalence of non-adherence in individuals who did not have an established place of care across all three risk factor groups (13.6% with no risk, 16.4% with one risk factor, and 19.9% with more than one risk factor).

### Adjusted odds ratio (OR) of CRMN (Table 2 *)*

**Table 2.** Factors affecting CRMN by CVD Risk factor status: Results from multivariate logistic regression model

	Cost-related non-adherence, OR [95% confidence interval (CI)]		
	No CVD risk factors	Any 1 CVD risk factor	>1 CVD risk factor
Age			
18–39	Reference	Reference	Reference
40–64	0.77 (0.65, 0.91)	0.70 (0.56, 0.87)	0.63 (0.44, 0.90)
≥65	0.31 (0.22, 0.43)	0.24 (0.17, 0.33)	0.22 (0.14, 0.34)
Sex			
Male	Reference	Reference	Reference
Female	1.48 (1.24, 1.76)	1.48 (1.25, 1.75)	1.59 (1.28, 1.98)
Ethnicity			
Non-Hispanic White	Reference	Reference	Reference
Non-Hispanic Black	1.47 (1.14, 1.90)	0.84 (0.65, 1.10)	1.17 (0.87, 1.56)
Non-Hispanic Asian	1.20 (0.84, 1.70)	0.68 (0.39, 1.18)	0.60 (0.33, 1.09)
Non-Hispanic all other race group	1.09 (0.76, 1.58)	0.56 (0.30, 1.04)	1.28 (0.79, 2.08)
Hispanic	1.08 (0.85, 1.36)	0.99 (0.76, 1.28)	0.91 (0.65, 1.27)
Educational status			
<High school	Reference	Reference	Reference
High school graduate	1.12 (0.86, 1.47)	0.92 (0.68, 1.24)	1.09 (0.80, 1.49)
Some college	1.43 (1.11, 1.83)	1.27 (0.94, 1.71)	1.51 (1.11, 2.06)
College graduate	1.29 (0.98, 1.69)	1.16 (0.84, 1.59)	1.44 (1.01, 2.05)
Income to poverty ratio			
<1	Reference	Reference	Reference
1–1.99	0.98 (0.79, 1.23)	0.96 (0.70, 1.30)	1.13 (0.84, 1.53)
≥2	0.49 (0.37, 0.64)	0.46 (0.33, 0.66)	0.52 (0.36, 0.76)
Marital status			
Unmarried	Reference	Reference	Reference
Married	1.11 (0.90, 1.38)	1.04 (0.81, 1.34)	1.34 (0.99, 1.80)
Region			
Northeast	Reference	Reference	Reference
Midwest	1.47 (1.18, 1.84)	1.09 (0.82, 1.46)	1.35 (0.95, 1.92)
South	1.41 (1.12, 1.77)	1.32 (1.01, 1.72)	1.53 (1.09, 2.15)
West	1.39 (1.10, 1.77)	1.03 (0.76, 1.39)	1.35 (0.93, 1.96)
Insurance			
Uninsured	Reference	Reference	Reference
Private	0.31 (0.25,0.39)	0.38 (0.28, 0.50)	0.39 (0.26, 0.59)
Medicaid	0.24 (0.18, 0.32)	0.24 (0.17, 0.35)	0.24 (0.15, 0.36)
Medicare	0.44 (0.29, 0.67)	0.48 (0.32, 0.71)	0.59 (0.38, 0.91)
Other public*	0.41 (0.10, 1.62)	<0.001 (<0.001, <0.001)*	0.13 (0.04, 0.45)
Smoking status			
Never smoker	Reference	Reference	Reference
Former smoker	1.48 (1.23, 1.77)	1.38 (1.12, 1.69)	1.23 (0.99, 1.51)
Current smoker	1.73 (1.42, 2.10)	1.78 (1.40, 2.26)	1.82 (1.40, 2.37)
BMI			
<18.5	Reference	Reference	Reference
18.5–24.9	1.66 (0.93, 2.95)	1.39 (070, 2.76)	2.79 (0.53, 14.58)
25–29.9	1.58 (0.89, 2.81)	1.57 (0.78, 3.16)	2.57 (0.49, 13.53)
≥30	1.72 (0.96, 3.07)	1.47 (0.73, 2.95)	2.76 (0.52, 14.58)
Self-rated health			
Good	Reference	Reference	Reference
Excellent	0.46 (0.36, 0.59)	0.45 (0.32, 0.63)	0.47 (0.27, 0.82)
Very good	0.70 (0.58, 0.84)	0.67 (0.54, 0.84)	0.70 (0.51, 0.95)
Fair	1.90 (1.48, 2.44)	1.96 (1.60, 2.41)	1.52 (1.20, 1.92)
Poor	1.88 (1.26, 2.79)	2.61 (1.85, 3.68)	2.94 (2.19, 3.96)
Count of adults in the household			
1	Reference	Reference	Reference
2	0.76 (0.62, 0.94)	1.06 (0.83, 1.36)	0.80 (0.59, 1.08)
3+	0.84 (0.67, 1.04)	1.07 (0.79, 1.44)	0.95 (0.69, 1.31)
Count of children in the household			
0	Reference	Reference	Reference
1	1.12 (0.89, 1.42)	1.03 (0.81, 1.31)	0.98 (0.69, 1.39)
2	0.90 (0.73, 1.12)	0.88 (0.64, 1.20)	0.89 (0.60, 1.34)
3+	1.02 (0.79, 1.32)	0.63 (0.42, 0.94)	1.44 (0.89, 2.34)
Usual place of care			
Yes	Reference	Reference	Reference
No	1.32 (1.02, 1.71)	1.43 (0.99, 2.08)	1.38 (0.83, 2.29)

*Unable to determine an accurate estimate as the analysis produced a considerably lower odds ratio and corresponding confidence interval.

In adjusted models, participants who were >65 years of age had lower odds of CRMN compared to 18–39 years across all three risk factor groups (Odds ratio of 0.31, 0.24, and 0.22 in no CVD risk factor, any one and more than one CVD risk factor groups respectively). Females with more than one CVD risk factor were 1.59 times more likely non-adherent than males (95% CI, 1.2–1.95). This was similar to the odds in the other two risk factor groups with 1.48 times in female participants with any one CVD risk factor (95% CI, 1.25–1.75) and 1.48 times in those without CVD risk factors (95% CI, 1.24, 1.76). There were no statistically significant associations between medication adherence and the various race/ethnicity among all three of CRMN groups.

Respondents who attended some college and had more than one CVD risk factor were 1.51 times more likely to be non-adherent (95% CI, 1.11–2.06) while those who had no risk factors were 1.43 times more likely to be non-adherent compared to people without high school education (95% CI, 1.11–1.83).

High-income families (ratio more than or equal to 2) has a lower odds of CRMN compared to low-income families (ratio less than 1) with an odds of 0.49 (95% CI, 0.37–0.64), 0.46 (95% CI, 0.33–0.66), and 0.52 (95% CI, 0.36–0.76) in no CVD risk factor, any one CVD risk factor and more than one CVD risk factor groups respectively. The odds of CRMN in people with any insurance were significantly less than the uninsured, with those on Medicaid who had the lowest odds of 0.24 in respondents across all three groups [no risk factors (95% CI, 0.18–0.32), any one (95% CI, 0.17–0.35), and more than one CVD risk factor (95% CI, 0.15–0.36) groups]. Among patients who had more than one CVD risk factor, participants from the South had the highest odds and were 1.53 times more likely to be non-adherent compared to the Northeast (95% CI, 1.09–2.15).

Current smokers with multiple CVD risk factors were 1.82 times more likely to be non-adherent compared to those who never smoked (95% CI, 1.40–2.37) with similar odds in the no CVD risk factor and respondents with any one CVD risk factor. In the same group, patients who had rated their health as poor were 2.94 times more likely to demonstrate higher CRMN than those who reported their health to be good (95% CI, 2.19–3.96) while those who rated their health as very good and excellent reported less CRMN (corresponding OR: 0.70, 95% CI, 0.51–0.95 and 0.47, 95% CI, 0.27–0.82).

### Association based on disease status (Table 3)

**Table 3.** Factors affecting CRMN by Disease status: Results from multivariate logistic regression model

	Cost-related nonadherence, OR (95% CI)		
	Hypertension	Diabetes	Hyperlipidaemia
Age			
18–39	Reference	Reference	Reference
40–64	0.85 (0.69, 1.06)	0.94 (0.67, 1.34)	0.79 (0.60, 1.03)
≥65	0.25 (0.19, 0.32)	0.31 (0.20, 0.48)	0.27 (0.19, 0.38)
Sex			
Male	Reference	Reference	Reference
Female	1.47 (1.26, 1.73)	1.30 (1.02, 1.64)	1.57 (1.30, 1.88)
Ethnicity			
Non-Hispanic White	Reference	Reference	Reference
Non-Hispanic Black	0.92 (0.74, 1.15)	0.89 (0.64, 1.24)	1.18 (0.91, 1.53)
Non-Hispanic Asian	0.55 (0.34, 0.89)	0.35 (0.17, 0.69)	0.67 (0.42, 1.07)
Non-Hispanic all other race group	0.99 (0.63, 1.57)	0.05 (0.48, 1.89)	1.32 (0.87, 1.98)
Hispanic	0.91 (0.71, 1.16)	0.64 (0.45, 0.91)	0.88 (0.67, 1.16)
Educational status			
<High school	Reference	Reference	Reference
High school graduate	0.83 (0.65, 1.07)	0.95 (0.66, 1.35)	0.98 (0.74, 1.30)
Some college	1.07 (0.84, 1.38)	1.24 (0.89, 1.72)	1.41 (1.08, 1.84)
College graduate	0.90 (0.69, 1.18)	1.18 (0.78, 1.76)	1.05 (0.78, 1.40)
Income to poverty ratio			
<1	Reference	Reference	Reference
1–1.99	0.93 (0.73, 1.19)	0.80 (0.57, 1.12)	0.98 (0.75, 1.27)
≥2	0.38 (0.28, 0.50)	0.39 (0.28, 0.58)	0.37 (0.27, 0.51)
Marital status			
Unmarried	Reference	Reference	Reference
Married	1.05 (0.90, 1.23)	1.12 (0.89, 1.42)	1.18 (0.98, 1.41)
Region			
Northeast	Reference	Reference	Reference
Midwest	1.34 (1.02, 1.74)	1.44 (0.99, 2.10)	1.24 (0.93, 1.65)
South	1.53 (1.20, 1.94)	1.60 (1.12, 2.28)	1.49 (1.14, 1.94)
West	1.27 (0.97, 1.68)	1.58 (1.05, 2.37)	1.13 (0.82, 1.56)
Insurance			
Uninsured	Reference	Reference	Reference
Private	0.39 (0.29, 0.51)	0.49 (0.33, 0.74)	0.30 (0.22, 0.43)
Medicaid	0.31 (0.22, 0.43)	0.26 (0.16, 0.41)	0.27 (0.18, 0.39)
Medicare	0.63 (0.46, 0.88)	0.75 (0.45, 1.24)	0.49 (0.34, 0.72)
Other public	0.10 (0.02, 0.42)	0.43 (0.09, 1.99)	0.09 (0.03, 0.31)
Usual place of care			
Yes	Reference	Reference	Reference
No	1.18 (0.81, 1.73)	1.98 (1.11, 3.52)	1.67 (1.10, 2.53)

**Figure 4.**
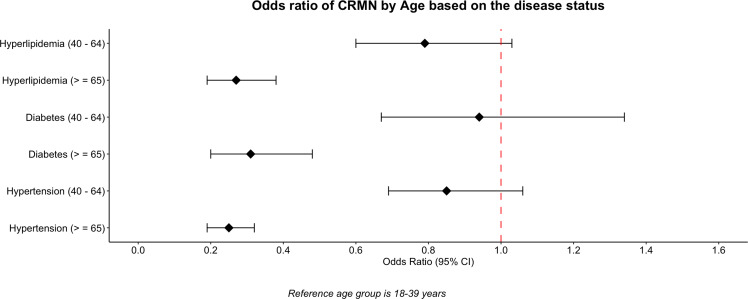
CRMN by Age based on the disease status.

**Figure 5.**
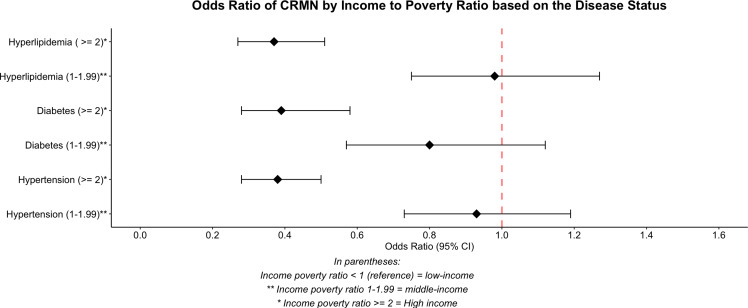
CRMN by Income to Poverty Ratio based on the disease status.

**Figure 6.**
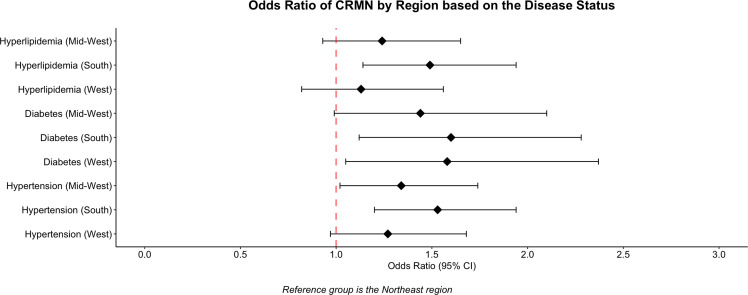
CRMN by Region based on the disease status.

After controlling for age, sex, ethnicity, educational status, income ratio to poverty, marital status, region, and insurance, participants were stratified based on their disease status, which included hypertension, diabetes, and dyslipidaemia. Patients who were more than 65 years of age with other public insurance or Medicaid had lower odds of non-adherence irrespective of whether they had hypertension, diabetes, or hyperlipidaemia (Figure 4). Similarly, respondents from high-income households also demonstrated lower odds of non-adherence across all the disease groups with an odds ratio of 0.38 (95% CI, 0.28–0.50) for hypertension, 0.39(95% CI, 0.28–0.58) for diabetes, and 0.37 (95% CI, 0.27–0.51) for hyperlipidaemia (Figure 5). CRMN, when compared to non-Hispanic white patients, was significantly less likely in non-Hispanic Asians who had hypertension (OR 0.55, 95% CI, 0.34–0.89) and diabetes (OR 0.35, 95% CI, 0.17–0.69). Further, females and those from the South had significantly higher odds of CRMN regardless of the disease category (Figure 6).

## Discussion

Our research reveals a prevailing pattern where patients with adverse social determinants of health, such as low-income, limited education, and insufficient access to healthcare resources, are significantly more prone to CRMN. Young respondents, unmarried individuals, females, those with less education, and participants in the South had a higher proportion of CRMN compared to older, married individuals, males, and those with higher education, residing in other regions. The comparison between individuals with and without CVD risk factors provided critical insight into how CRMN varies by clinical conditions and risk factors. The observed differences highlight the need for condition-specific strategies while also framing CRMN as a broader systemic issue. These findings emphasize the importance of addressing CRMN through both population-wide interventions and targeted efforts for clinically vulnerable groups. Additionally, they highlight the significant role of financial and social barriers in shaping access to medication and adherence, with substantial implications for overall health outcomes.

Our results suggest that non-adherence was lowest in participants over 65 years of age among all three risk factor groups (3.4% in participants with no CVD risk factors, 3.6% in those with one CVD risk factor, and 4.4% among individuals with more than one CVD risk factor). (Figure 1) This could be explained by the rise in the number of comorbid diseases in older individuals, increasing health awareness with age, greater resource allocation towards medications, and better prescription drug benefits like the Medicare Part D programme (Zhang *et al.*, 2022: 2004–2014; ‘NHIS - About the National Health Interview Survey’, 2023).

Our analysis also determined that females with more than one CVD risk factor had higher odds of being non-adherent compared to males (OR 1.59, 95% CI, 1.28–1.98). Although previous evidence shows that women are more likely to seek care compared to men, some studies suggest that women as primary caregivers prioritize their family’s health over their own (Rolnick *et al.*, 2013; Thompson *et al.*, 2016; Höhn *et al.*, 2020). Additionally, an increasing financial burden based on the number of dependent children could also significantly contribute towards lower medication adherence in females (Rolnick *et al.*, 2013; Zhang *et al.*, 2017). Additional research reveals that women are more prone to medication side effects, such as statin-induced myopathy, and have higher rates of depressive disorders. Moreover, women often receive substandard care, encounter lower levels of social support, and demonstrate lower awareness of disease risks (Bird *et al.*, 2007; Nekui *et al.*, 2021; Venditti *et al.*, 2023). This highlights the importance of developing better healthcare policies for younger adults not covered by Medicaid and women to facilitate reducing the disparities that are associated with CRMN (Zhang *et al.*, 2022).

Among all the risk factor groups, higher proportions of CRMN were found among respondents who received less than a high school level of education followed by those with some form of college education (Lee and Khan, 2016). The former may face significant strain with a higher chance of being unemployed or failing to maintain a steady income (‘Trends in High School Dropout and Completion Rates in the United States’, 2024). Either situation can cause financial strain and affect their insurance status as adults under 65 are primarily dependent on their employer-based insurance thereby impacting their medication adherence (Oates *et al.*, 2020; Nekui *et al.*, 2021; ‘Trends in High School Dropout and Completion Rates in the United States’, 2024). In a cross-sectional analysis of the NHIS data between 2010–2015, Su, Chia-Ping et al. determined that people with non-standard jobs like freelance work, temporary contract-based employment, and working in small establishments were more likely to be uninsured (Su *et al.*, 2019). Our findings suggest that a robust social safety net that includes drug benefits might help mitigate the risk of CRMN among low-income and lowereducation groups.

Studies have found that poor economic status and underlying disease were negatively associated with self-rated health (Basirimoghadam *et al.*, 2020; Cialani and Mortazavi, 2020). The inability to access health resources due to financial hardship aggravates psychological distress and causes deterioration of overall well-being, making it more challenging to afford medications (Tucker-Seeley *et al.*, 2013). This may explain our findings that respondents from high-income households were more likely to be adherent compared to lower-income families irrespective of their disease status or the risk factor groups. Our study also determined that, within all risk factor groups, participants with three or more children had significantly higher non-adherence rates than those with no children. Studies found that families with children and in particular, low-income families are unable to bear the burden of insurance and out-of-pocket healthcare costs, which prove detrimental to their health needs (Wisk and Witt, 2012; Dong *et al.*, 2021).

In our assessment, although ethnicity was not a notable contributing factor toward medication adherence, there were significant regional differences observed across the country. Individuals from the South with more than one CVD risk factor were 1.59 times more likely than those from the Northeast to experience CRMN. It is prudent to note that some of the states in the Southern region of the U.S. are among the few that have chosen not to expand their Medicaid. According to the Centres for Disease Control, the residents of the Medicaid non-expansion states were more likely to be uninsured as they are neither eligible for Medicaid nor for other subsidized insurance coverage plans if their income is in the low to moderate range which would potentially affect medication adherence among these populations (Heller *et al*., 2023; Terlizzi and Cohen, 2022).

Besides insurance, the regional variation in adherence can also be affected by factors like polypharmacy and complex drug regimens involving taking multiple pills at different times of the day, poor access to pharmacies especially in rural areas, and higher out-of-pocket expenses in areas with predominantly low-income populations (Yang, 2020). These can be rectified by introducing better prescription practise guidelines involving generic fixed-dose drug combinations which will reduce the costs as well as the number of pills, increasing doorstep delivery of medications by switching to mail-order or online pharmacy practises (Jimmy and Jose, 2011; Marcum *et al*., 2014).

Our results also revealed that CRMN was higher among current smokers compared to non-smokers. This may be directly linked to the greater expenditure of their annual income on tobacco in comparison to medication (Lal *et al*., 2022). Additionally, in those classified as obese with a BMI of greater than 30, CRMN was significantly higher than in those with lower BMIs. Previous studies propose that weight-based bias perpetuated by the community has possibly led to less desire and motivation to seek care by overweight or obese people. Similarly, physician perceptions that obese patients are less likely to be adherent can further reinforce the bias by influencing the management and treatment of these. Interestingly, an individual’s annual medical care costs increase significantly with an increase in the class of obesity, going from 68.4% in class 1 to 233.6% in class 3 which can be due to a greater number of comorbidities in obese patients (Cawley *et al*., 2021).

Medications for cardiovascular diseases and their risk factors represent a significant portion of annual spending. Recent years have seen the approval of costly, patented drugs like angiotensin receptor-neprilysin inhibitors (ARNIs) and PCSK9 inhibitors. The Inflation Reduction Act of 2022 aims to address this by capping insulin costs at $35/month, providing free adult vaccines, limiting out-of-pocket expenses to $2000 annually, allowing Medicare to negotiate drug prices, and requiring rebates from drug companies for rapid price increases (Birger *et al*., 2021; ‘Inflation Reduction Act and Medicare | CMS’, n.d.; Varghese *et al*., 2021). While this landmark law represents a significant step, ongoing research is required to evaluate the impact and effectiveness on drug affordability and accessibility, especially due to the limited Medicare expansion in a few southern states. With rising healthcare costs directly attributed to the struggle to afford medication, it is also imperative to strategize and promote healthy habits (Shrank *et al*., 2021). Lee *et al.* demonstrated that lifestyle modifications including cessation of smoking and a weight-loss diet were statistically associated with medication adherence, indicating the importance of interventions such as motivational education programmes (Lee *et al*., 2018). These features are potential modifying factors that are attributable to CRMN.

These results have important implications for health policy. Expanding Medicaid in non-expansion states, streamlining prior authorization procedures, and incorporating risk-based flags into electronic health records may help identify individuals at risk for CRMN (Cover Story | Affordability, 2018; Jew *et al*., 2020). Policies that cap prescription drug prices, enable bulk purchasing, and incentivize generic alternatives could alleviate cost burdens (‘340B Drug Pricing Program | HRSA’, n.d.; Clark and Puthiyath, 2022; Gaffney *et al*., 2020). Furthermore, promoting community-based outreach, motivational education programmes, and lifestyle medicine interventions may enhance adherence, particularly among those facing economic and social barriers. As health systems and policymakers work toward improving population health, addressing CRMN must remain a central priority.

## Strengths and limitations

Our study is strengthened by the sample size. Furthermore, NHIS data are nationally representative, and hence the estimates are generalizable to the US population. Our study is also the first to use nationally representative data to examine the prevalence and factors associated with CRMN among people with and without CVD risk factors. However, the study has a few limitations. One of the reasons for lower response rates in 2020 and 2021 compared to 2019 was challenges during the COVID-19 pandemic, with restrictions on face-to-face data collection and hesitancy of respondents to participate during the pandemic. Since the data are self-reported, they may have been subject to recall bias, social desirability bias, and other measurement biases. Finally, the data is cross-sectional, limiting its use for causal inference and observing temporal relationships between various health factors.

## Conclusion

Our study revealed variation in CRMN among the U.S. population with and without cardiovascular risk factors. Younger adults, females, unmarried individuals, those with lower education levels, and residents of the South had significantly higher odds of CRMN. A lack of public or private insurance coverage emerged as one of the key predictors of CRMN, contributing to disparities across all groups. Many of the disparities observed may, in fact, reflect underlying differences in access to health insurance. These findings highlight the importance of prioritizing populations at risk when developing strategies to enhance medication adherence and mitigate cost barriers. Further research is warranted to identify effective interventions that address these gaps and ensure equitable access to essential medications.
